# The Influence of **α**-Lipoic Acid and Garlic Administration on Biomarkers of Oxidative Stress and Inflammation in Rabbits Exposed to Oxidized Nutrition Oils

**DOI:** 10.1155/2015/827879

**Published:** 2015-11-08

**Authors:** Jolanta Zalejska-Fiolka, Tomasz Wielkoszyński, Wojciech Rokicki, Natalia Dąbrowska, Joanna Katarzyna Strzelczyk, Aleksandra Kasperczyk, Aleksander Owczarek, Urszula Błaszczyk, Sławomir Kasperczyk, Barbara Stawiarska-Pięta, Ewa Birkner, Andrzej Gamian

**Affiliations:** ^1^Department of Biochemistry, School of Medicine with the Division of Dentistry in Zabrze, Medical University of Silesia in Katowice, Jordana 19, 41-808 Zabrze, Poland; ^2^Clinic of Ophthalmology, School of Medicine in Katowice, Medical University of Silesia in Katowice, Ceglana 35, 40-952 Katowice, Poland; ^3^Department of Medical and Molecular Biology, School of Medicine with the Division of Dentistry in Zabrze, Medical University of Silesia in Katowice, Jordana 19, 41-808 Zabrze, Poland; ^4^Department of Statistics, SPLMS in Sosnowiec, Ostrogórska 30, 43-302 Sosnowiec, Poland; ^5^Department of Pathology, SPLMS in Sosnowiec, Ostrogórska 30, 43-302 Sosnowiec, Poland; ^6^Department of Medical Biochemistry, Wrocław Medical University, Chałubińskiego 10, 50-368 Wrocław, Poland

## Abstract

We hypothesized that addition of substances with antioxidant activity could decrease the concentrations of biomarkers of oxidative stress and inflammatory process, thus inhibiting nonalcoholic steatohepatitis development. We investigated the influence of *α*-lipoic acid (ALA) and garlic administration on the development of adverse changes in rabbit liver and serum under oxidative stress conditions induced with HFD from oxidized oils. We determined 8-hydroxy-2′-deoxyguanosine (8OHdG) and malondialdehyde (MDA) in liver homogenates, total oxidant status (TOS), lipid peroxides (LOO) and tumor necrosis factor alpha (TNF*α*) in blood serum, and TNF*α* and IL-1*α* genes expression in liver. The results indicate that the intake of dietary ALA and garlic was significantly associated with decreases of 8OHdG and MDA levels in rabbits' liver tissue as well as TOS and LOO levels in rabbits' serum. Similarly, TNF*α* and IL-1*α* gene expressions were suppressed due to ALA and garlic supplementation. The histopathological analysis confirmed that HFD results in liver disorder leading to steatosis. This adverse effect of HFD was ameliorated by the supplementation of ALA and garlic. The obtained results indicate a beneficial effect of ALA and garlic administration by reducing the oxidative stress intensity and the levels of some proinflammatory cytokines in rabbits fed HFD.

## 1. Introduction

Fat-rich diets and the consumption of oxidized oils are factors that result in increased concentrations of reactive oxygen species (ROS), negatively impacting the pro- and antioxidative equilibrium. Such oxidative stress and disruption of homeostasis damage proteins, DNA, and lipids. Lipid peroxidation is recognized as one of the most prominent consequences of increased generation of free radicals. It is a multistage, uncontrolled process that involves polyunsaturated fatty acids (PUFA), leading to generation of substantial amounts of noxious products such as lipid- and peroxylipid radicals, coupled or conjugated dienes and trienes, and peroxides and hydroxyl-peroxides of fatty acids (FA). Low-molecular-weight products of lipid degradation, such as malondialdehyde (MDA) and 4-hydroxynonenal (4HNE), have been extensively used as markers of oxidative stress [1–4].

In recent years other indicators of protein and DNA lesions caused by free radicals have largely attracted the attention of researchers [5–8]. Oxidation products of proteins such as guanine, guanosine, and deoxyguanosine turn out to be more stable and their determination seems to be more specific in the course of oxidative stress. Among these products 8OHdG stands out as biomarker of oxygen-related lesions of DNA and cellular oxidative stress [9–13], whose mutagenic potential results from erroneous base pairing during DNA replication [11]. Research has also been conducted to find a correlation between the amounts of 8-hydroxy-2′-deoxyguanosine (8OHdG) in various tissues with pathogenic processes. Elevated 8OHdG has also been detected in patients suffering from atherosclerosis, diabetes, neurodegenerative diseases, or autoimmune diseases [14–17].

Oxidative stress and inflammation induced by a high-fat diet plays an important role in the development of steatohepatitis, so an HFD may hasten the development of nonalcoholic steatohepatitis (NASH).

A positive factor in this regard is the use of foods having antioxidative properties. New substances are continuously searched for, such as *α*-lipoic acid (ALA), which helps to modulate the oxidation-reduction processes in the organism [18, 19]. Recent studies have demonstrated that regular intake of ALA helped as a preventive and/or coadjuvant in the treatment of many diseases such as diabetes and concomitant diseases, diseases of the cardiovascular or neurological systems, and those derived from viral infections [18–20]. In this regard, there is an ongoing discussion about the potential role of ALA as a therapeutic agent in diseases of the liver [21–25].

Garlic has also been reported as a natural antioxidant. It has played an important dietary and medicinal role throughout the history of mankind. The therapeutic efficacy of garlic encompasses a wide variety of ailments that include cancer, hepatic and microbial infections, and cardiovascular diseases. As ROS seem to be at the core of many ailments, it is justified to assume that the beneficial effects of garlic might be through modulation of ROS [26].

Taking into account the wide spectrum of actions of these substances, it was decided to study their influence on peroxidation of lipids and proteins in animals fed with oxidized vegetable oils.

## 2. Materials and Methods

### 2.1. Preparation of Oil Samples

Rapeseed and olive oils were purchased from a local supermarket. For its oxidation, one-liter oil was heated to a temperature of 180°C for a period of six hours. The extent of oxidation was determined by measuring the peroxide and iodine levels, as suggested by the Polish Norm Standardization Committee [27]. The content of FA was measured by a literature chromatographic method [18].

### 2.2. Animals

The present study lasted twelve weeks. It was conducted following the Guidelines of the Animal Care Committee of the University of Silesia. It was assigned the approval number KNW-022/LKE-1-25/08.

Fifty-four male Chinchilla rabbits (b.m. 2800 ± 200 g) were obtained from the Center for Experimental Medicine, Medical University of Silesia in Katowice. The animals were housed individually in stainless steel metabolic cages under a 12-hour light/dark cycle. The rabbits were fed 80 g proper fodder per kg b.m. once a day, allowing free access to water. The animals were weighed weekly, throughout the duration of the experiment, evaluating the leftover food debris to assess feed intake. The amount of additive given to each rabbit was calculated according to the weight of the animal and the modified duration of the experiment.

The rabbits were divided into nine groups of six animals each, according to the following scheme:

1. controls (C): rabbits fed only a basal diet (BD),
2. oxidized olive oil group (OO): rabbits fed BD with 10% oxidized olive oil added,
3. oxidized rapeseed oil group (RO): rabbits fed BD with 10% oxidized rapeseed oil added,
4. *α*-lipoic acid group (A): rabbits fed BD with 10 mg/kg b.m. *α*-lipoic acid added,
5. garlic group (G): rabbits fed BD with 4 mg/kg b.m. standardized garlic extract added,
6. oxidized olive oil with *α*-lipoic acid group (OOA): rabbits fed BD with 10% oxidized olive oil and 10 mg/kg b.m. *α*-lipoic acid added,
7. oxidized rapeseed oil with *α*-lipoic acid group (ROA): rabbits fed BD with 10% oxidized rapeseed oil and 10 mg/kg b.m. *α*-lipoic acid added,
8. oxidized olive oil with garlic group (OOG): rabbits fed BD with 10% oxidized olive oil and 4 mg/kg b.m. standardized garlic extract added,
9. oxidized rapeseed oil with garlic group (ROG): rabbits fed BD with 10% oxidized rapeseed oil and 4 mg/kg b.m. standardized garlic extract added.

The specific diets for each group were prepared weekly and stored in a refrigerator at 4°C. The BD was composed of 24% protein, 69% carbohydrate, and 7% fat of the total energy content of the diet. Groups fed BD with 10% oxidized oils received, respectively, 18% energy from protein, 39% from carbohydrate, and 42% from fat.

### 2.3. Sample Collection

At the end of the study period the rabbits were sacrificed under anesthesia with a mixture of 50 mg/kg-bm ketamine, 0.1 mg/kg-bm fentanyl, and 0.1 mg/kg-bm droperidol administered by intramuscular injection following a 12 h fasting. The livers were collected for biochemical study after isotonic saline rinsing.

### 2.4. Biochemical Examination

#### 2.4.1. The Concentration of TOS and LOO

The concentrations of total oxidant status (TOS) and lipid peroxides (LOO) in blood serum were measured according to Erel [28] and Södergren et al. [29] methods, respectively, using automated analyzer VICTOR-X3 Perkin Elmer calibrated with hydrogen peroxide. Data were shown in *μ*mol/L.

#### 2.4.2. The Concentration of TNF*α*

The concentration of TNF*α* was measured in blood serum by ELISA method using goat anti-rabbit TNF*α* antibody as capture antibody and biotinylated, monoclonal anti-rabbit TNF*α* antibody as tracer (both from BD Pharmingen, USA). The assay was performed according to the manufacturer's instruction: goat antibody was immobilized on ELISA plates (Maxisorp, Nunc, Denmark) and bovine serum albumin was used for blocking of unbound sites. Standard curve was constructed with the use of rabbit TNF*α* (0,05–10 ng/mL; BD Pharmingen, USA) in BSA solution. Serum samples and TNF*α* standards were incubated for 2 hours and washed out with PBST. Next, biotinylated anti-TNF*α* was incubated for 1 hour. Immobilized immunocomplexes were detected with streptavidin-horseradish peroxidase conjugate (Dako-Cytomation, Denmark; 30 minutes) and visualized using TMB Supersensitive System (Sigma-Aldrich, USA). Then, the reaction was stopped with 0,5 M sulfuric acid. The absorbance was measured on PowerWave XS ELISA plate reader (BioTek, USA; 450 nm/630 nm as reference). KCJunior (a computer program) (BioTek, USA) was used to collect data. Results were presented as pg of TNF*α* per mL of serum [pg/mL]. Interassay error was 6.4%.

#### 2.4.3. The Concentration of MDA

The concentration of MDA was determined in liver homogenate by the method of Ohkawa et al. [30]. Samples of the liver homogenates (10% in saline) were mixed with 8.1% sodium dodecylsulphate, 20% acetic acid, and 0.8% 2-thiobarbituric acid. After vortexing, the samples were incubated for 1 h at 95°C and then butanol-pyridine 15 : 1 (v/v) was added. The mixture was shaken for 10 min and then centrifuged. The butanol-pyridine layer was measured fluorometrically at 552 nm (515 nm excitation). Thiobarbituric acid reactive substances (TBARS) values are expressed as MDA equivalents. Tetraethoxypropane was used as a standard. Data are reported as *μ*mol/mg of protein. The concentration of protein was determined according to the method of Lowry et al. [31].

### 2.5. Isolation of DNA for Determination of 8OHdG

The level of 8OHdG and 2′-deoxyguanosine (2′dG; for 8OHdG calculation) was determined in isolated DNA using high-pressure liquid chromatography (HPLC) coupled with an electrochemical detector for 8OHdG and with a UV detector for detection of 2′-dG. The samples were prepared in the following sequence. First, DNA was extracted from a fragment of liver (ca. 300 mg) surgically obtained at dissection. Then, the tissue fragments were homogenized using Lysing Matrix D (MP Biomedicals, USA) on a FastPrep-24 homogenizer (MP Biomedicals, USA). Total DNA was extracted using a commercial kit GeneMATRIX Tissue DNA Purification Kit (EURx, Poland) following the manufacturer's instructions. The DNA concentration was measured using a ND-1000 spectrophotometer (Thermo Scientific, USA). Subsequently DNA was hydrolyzed to nucleotides. The samples were prepared based on the method published by Foksinski et al. [32]. To 100 *μ*L of the isolated DNA was added 50 *μ*L of 40 mM sodium acetate pH 5.1 buffer (POCH, Poland), 5 *μ*L of 0.1 mM zinc chloride (Merck, Germany), and 10 *μ*L of nuclease P1 (20 *μ*g protein per sample; Sigma-Aldrich, USA) and incubated for one hour at 37°C. Next, 15 *μ*L of 1 M Tris-HCl buffer, pH 7.4 (Fluka, USA), and 5 *μ*L of alkaline phosphatase (1.5 U, Sigma-Aldrich, USA) were added to remove the phosphate residues of the received nucleotides and then incubated for one hour at 37°C. The enzymes were removed from the sample using a membrane filter Amicon Ultra-4 (Millipore, USA) by centrifugation at 8000 rpm for 40 min. The prepared samples were loaded onto a chromatographic column.

Standard solutions of 8OHdG (Sigma-Aldrich, USA) at concentrations of 62.50 ng/mL, 31.25 ng/mL, 15.62 ng/mL, 7.81 ng/mL, 3.91 ng/mL, 1.95 ng/mL, 0.98 ng/mL, and 0.49 ng/mL were prepared using the mobile phase as solvent. The standard solutions of 2′dG (Fluka, USA) were prepared in the same way.

The following conditions were used for analytical procedures: the liquid chromatograph (KNAUER) coupled with an HPLC pump (K-1001 KNAUER) and two detectors: one for UV/VIS (KNAUER) and a Recipe Amperometric Detector EC300 (KNAUER). The measurements cell had three electrodes: a silver/silver chloride reference electrode, a working glassy carbon in zirconium oxide electrode, and a stainless steel auxiliary electrode. Data processing was done using EuroChrom 2000. HPLC separation was performed on a reverse phase Eurospher II 100-5 C18 column (KNAUER KU1010), 125 mm × 4 mm with precolumn following the method of Shigenaga et al. [33]. The mobile phase consisted of 50 mM, pH 5.5 K_2_HPO_4_, and methanol (9 : 1 v/v). 20 *μ*L of samples was injected to the column. Elution was carried out in isocratic mode at a flow rate of 0.7 mL/min. 8OHdG was determined at 30°C. The wavelength for determination of 2′dG was that of maximum absorbance. The cell potential was set at +0.6 mV. The content of 8OHdG was calculated as the ratio of concentrations of 8OHdG to 2′dG read from the corresponding calibration curves.

### 2.6. RNA Extraction and RT-PCR

Immediately after surgery, the specimens (fragments of rabbits' livers) were submerged in the tissue storage and RNA stabilization solution RNAlater (Sigma-Aldrich, USA) and stored at −80°C. Samples (using ceramic beads Lysing Matrix D (MP Biomedicals, USA)) were homogenized using homogenizer FastPrep-24 (MP Biomedicals, USA). RNA was isolated using the RNeasy Mini Kit (Qiagen, Germany). To remove residual genomic DNA, DNase I digestion was performed (RNase Free DNase Set, Qiagen, Germany). RNA was quantified by measuring the UV absorbance at 260/280 nm (NanoDrop ND-1000 Spectrophotometer, Thermo Fisher Scientific, USA). The Agilent Bioanalyzer 2100 (Agilent Technologies, USA) using Agilent RNA 6000 Nano Kit (Agilent Technologies, USA) allowed assessing the RIN (RNA Integrity Number) of previously isolated RNA. Subsequently, total RNA from each sample was reverse-transcribed into cDNA using High Capacity cDNA Reverse Transcription Kit with RNase Inhibitor (Applied Biosystems, USA) according to the manufacturers' instructions.

### 2.7. Expression of the Selected Genes (*TNFα* and* IL-1α*) Using qRT-PCR

Genes expression levels were analyzed by quantitative reverse transcription (qRT)-PCR using TaqMan Gene Expression Master Mix and specific TaqMan Gene Expression Assays (Applied Biosystems by Life Technologies, USA) for* IL-1α*, interleukin 1 alpha (Oc03399253_m1), and* TNFα*, tumor necrosis factor alpha (Oc03397715_m1), genes. All reactions were carried out using the 7300 Real-Time PCR System (Applied Biosystems, USA) according to the ABI recommended chemical and cycling conditions and analyzed by SDS 1.4 software (Applied Biosystems, USA). All assays were performed in triplicate. The* GAPDH*, glyceraldehyde-3-phosphate dehydrogenase gene (Oc03823402_g1), was used as internal control for normalization of the gene expression levels. The comparative threshold cycle (Ct) method 2^−ΔΔCt^ was used to determine the relative gene expression levels (RQ) for each of the target genes. Five samples from control group (5 livers) were used as a calibrator. Relative mRNA expression was determined from all examined samples using mRNA expression from calibrator. Thus, RQ reflect the ratio between the expression of target genes in the samples and in the calibrator.

### 2.8. Tissue Examination

The tissues needed for microscopic examination were obtained right after the animals were sacrificed. The specimens were collected from the right lobe of the liver and then divided into two parts, one of which was fixed in 10% formalin in PBS for morphological examination.

The presence of morphological lesions was assessed using a standard paraffin technique. Paraffin blocks were cut into sections with a thickness of 4 *μ*m using a sliding microtome (Leica SM 2000R), washed, and stained by the standard H-E method [34].

The histopathological examination was done under an Olympus light microscope. Microphotographs were taken using a digital camera (CAMEDIA C-3040; Olympus).

### 2.9. Statistical Data Analysis

Statistical analysis was performed using STATISTICA 10.0 PL (StatSoft, Cracow, Poland). The data were expressed as mean value ± standard deviation. All tests were two-tailed, setting the significance level at *P* < 0.05. Distribution of variables was evaluated by the Shapiro-Wilk test, and homogeneity of variances was assessed by the Levene test. In order to compare the MDA and 8OHdG liver level and TOS, LOO, and TNF*α* serum level and* TNFα*,* IL-1α* gene expression two-way analysis of variances was used, with Tukey's RIR post hoc test.

## 3. Results

### 3.1. Oxidized Oils

Table 1 gives the percentage content of fatty acids (FA) in the oxidized oils used in this study. The peroxide (PV) and iodine (IV) values determined in oils before and after oxidation are presented. Oils oxidized for 6 h at 180°C showed increased levels of palmitic and oleic acids and decreased contents of linolic and linolenic acids. Oxidized rapeseed oil showed a 106-fold PV increase and a decrease of its IV by about 2%. Olive oil oxidation increased PV by 18% and a decrease of 3% of the IV occurred.

### 3.2. Concentrations of Oxidative Stress Biomarkers and Protein Associated with Inflammation

**Figure 1:**
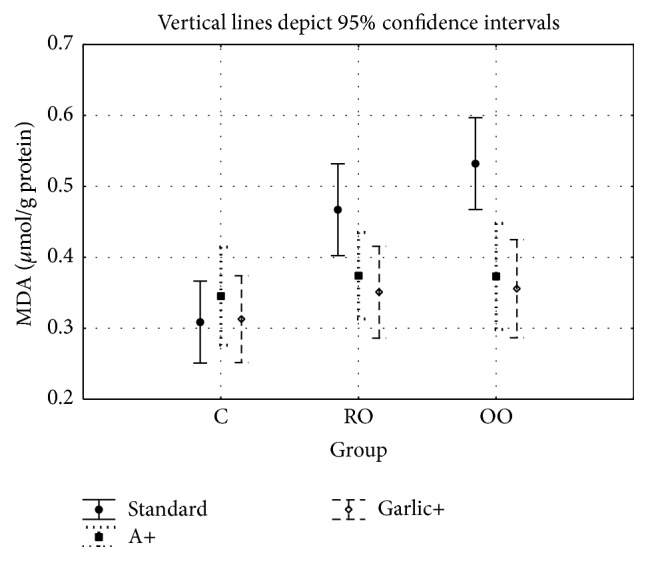
Malondialdehyde concentrations in the liver of rabbits in all study groups.

**Figure 2:**
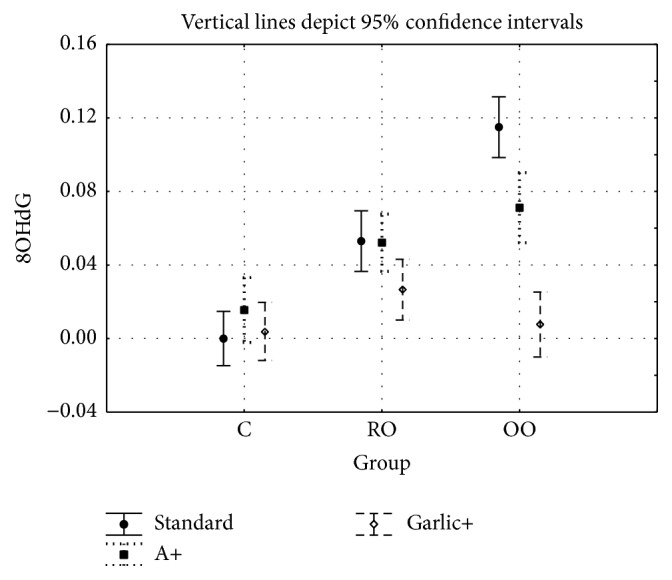
Level of 8-hydroxy-2′-deoxyguanosine in DNA of rabbits in all study groups.

Figures 1 and 2 give the concentrations of MDA and 8OHdG determined in rabbits' liver, respectively. The results were obtained at the end of the experimental period in liver homogenates from all experimental groups. Values are given as the means ± SD. For MDA concentration, the two-factor analysis of variance showed a significant main effect for the group type (C versus RO versus OO: *F* = 7.68, *P* < 0.01), a significant main effect for the treated groups (C versus A versus garlic: *F* = 7.46, *P* < 0.01) and a significant interaction between group type and treatment (*F* = 3.21, *P* < 0.05).

For the 8OHdG level, the two-factor analysis of variance showed a significant main effect for the group type (C versus RO versus OO: *F* = 38.51, *P* < 0.001), a significant main effect for treated groups (C versus A versus garlic: *F* = 23.59, *P* < 0.001) and a significant interaction between group type and treatment (*F* = 12.74, *P* < 0.001).  Table 2 presents the statistics and Table 3 gives the post hoc tests.

The MDA concentration in liver tissue showed no significant differences between the control and ALA and garlic groups. Significant differences are seen between the OO and OOA (*P* < 0.001) groups and between the OO and OOG groups (*P* < 0.001), while there are no significant differences between the OOA and OOG groups. Also, no significant differences were found between the RO and ROA groups or between the RO and ROG groups.

There were no significant differences for the 8OHdG level in liver tissue of controls, A, and garlic groups, but differences between the OO and OOA groups (*P* < 0.001), the OO and OOG groups (*P* < 0.001), and the OOA and OOG groups (*P* < 0.001) were significant. The differences between the RO and ROG groups and between the ROA and ROG groups were also significant (*P* < 0.05). There was no statistically significant difference between the RO and ROA groups.

**Figure 3:**
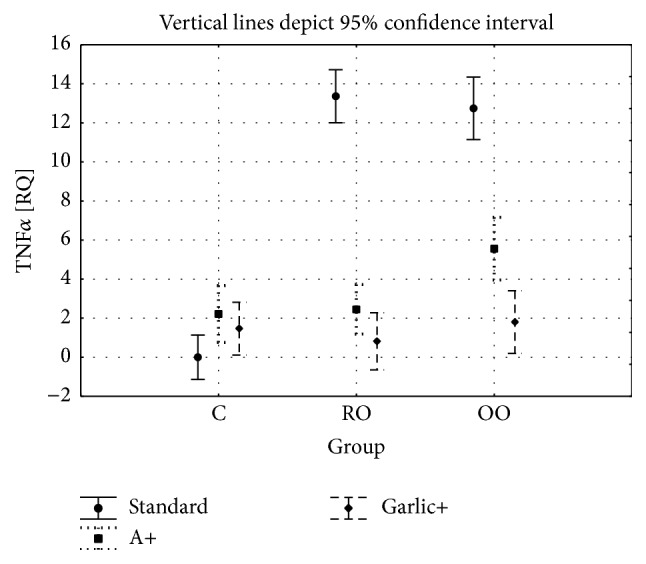
TNF*α* gene expression in all study groups.

**Figure 4:**
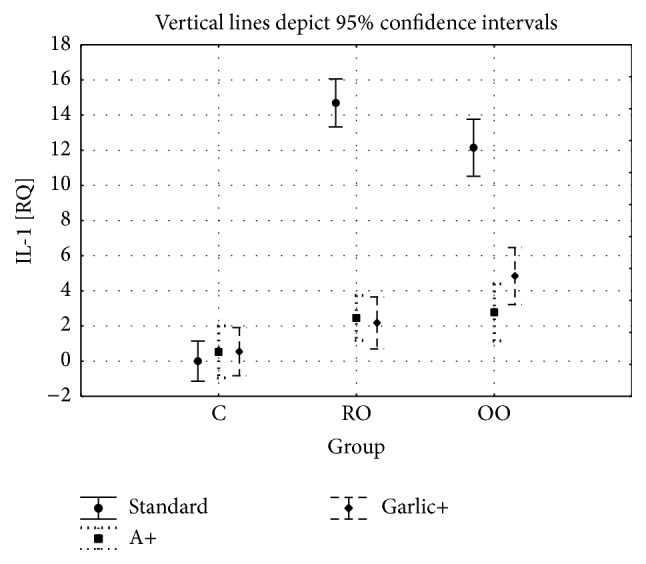
IL-1*α* gene expression in all study groups.

Figures 3 and 4 show the RQ of TNF*α* and IL-1*α* determined in liver homogenates of rabbits from all experimental groups. The results were obtained at the end of the experimental. Values are given as the means ± SD. For the TNF*α* gene expression, the two-factor analysis of variance showed a significant main effect for the group type (C versus RO versus OO: *F* = 51.2, *P* < 0.001), a significant main effect for the treated groups (C versus A versus garlic: *F* = 86.0, *P* < 0.001), and a significant interaction between group type and treatment (*F* = 42.8, *P* < 0.05).

For the IL-1*α* gene expression, the two-factor analysis of variance showed a significant main effect for the group type (C versus RO versus OO: *F* = 79.0, *P* < 0.001), a significant main effect for the treated groups (C versus A versus garlic: *F* = 90.7, *P* < 0.001), and a significant interaction between group type and treatment (*F* = 34.7, *P* < 0.05).

Table 4 presents the statistics and Table 5 gives the post hoc tests for TOS, LOO, and TNF*α* levels determined in blood serum of rabbits from all experimental groups. The results were obtained at the end of the experimental period. Values are given as the means ± SD.

For the TOS concentration, the two-factor analysis of variance showed a significant main effect for the group type (C versus RO versus OO: *F* = 9.70, *P* < 0.001), a significant main effect for the treated groups (C versus A versus garlic: *F* = 19.09, *P* < 0.001), and a significant interaction between group type and treatment (*F* = 3.34, *P* < 0.05). For the LOO concentration, the two-factor analysis of variance showed a significant main effect for the group type (C versus RO versus OO: *F* = 62.9, *P* < 0.001), a significant main effect for the treated groups (C versus A versus garlic: *F* = 16.01, *P* < 0.001), and a significant interaction between group type and treatment (*F* = 11.32, *P* < 0.001). For the TNF*α* concentration, the two-factor analysis of variance showed a significant main effect for the group type (C versus RO versus OO: *F* = 32.89, *P* < 0.001), a significant main effect for the treated groups (C versus A versus Garlic: *F* = 4.34, *P* < 0.05), and a significant interaction between group type and treatment (*F* = 4.61, *P* < 0.01).

The TOS and LOO concentrations in blood serum showed no significant differences between the control and ALA and garlic groups. Significant differences were shown between the OO and OOA (*P* < 0.001) and RO and ROA (*P* < 0.01) groups and between the OO and OOG (*P* < 0.001/*P* < 0.01) and RO and ROG (*P* < 0.01/*P* < 0.001) groups, while there were no significant differences between the OOA and OOG groups. The TNF*α* concentration in blood serum showed significant differences between the control and the garlic groups (*P* < 0.05). There were no differences between the OO and OOA groups (*P* = 0.474) as well as between the RO and ROA (*P* < 0.065) groups. At the same time, significant differences were shown between the OO and OOG groups (*P* < 0.05) as well as between the RO and ROG groups (*P* < 0.05). There were no significant differences between the OOA and OOG and ROA and ROO groups. The mRNA gene expression of* TNFα* and* IL-1α* showed a similar direction of change. Significant differences were shown between the OO and OOA, OO and OOG, RO and ROA, and RO and ROG groups, while there were no differences between the OOA and OOG and ROA and ROG groups. We observed a significant difference between the OOA and OOG groups for IL-1*α* gene expression, while there were no changes between the ROA and ROG groups.

### 3.3. Histological Assessment of the Liver

The liver of rats fed oxidized oils showed macroscopic changes such as enlargement and color changes in the liver, which were more evident in the OO group. The livers of all animals in that group were brighter and slightly xanthochromic compared to livers of animals from all other study groups, including controls. Similar changes, yet less intense, were detected in three rabbits that received RO.

### 3.4. Microscopic Examination of Liver Sections

**Figure 5:**
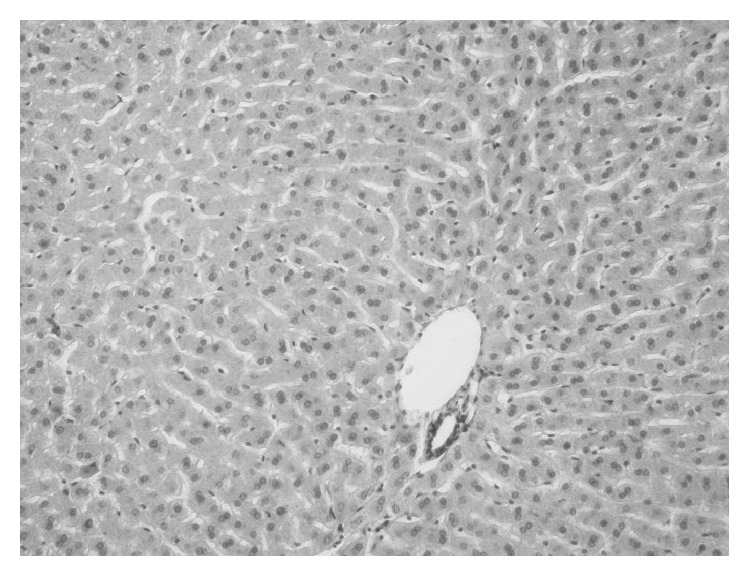
Liver tissue microphotography of controls: there are no abnormal changes of hepatocytes. H-E staining, 200x.

No changes were seen in livers of controls and from groups that received *α*-lipoic acid and garlic. As shown in Figure 5, tissues from lobule and stroma have normal appearance. No fatty degeneration or necrosis was observed in the ALA group, except in two rabbits that had a slight infiltration of mononuclear cells.

In the groups that received oxidized oils, the lesions were similar in character, but their intensity depended on the oil used. They were most pronounced in animals receiving no *α*-lipoic acid.

**Figure 6:**
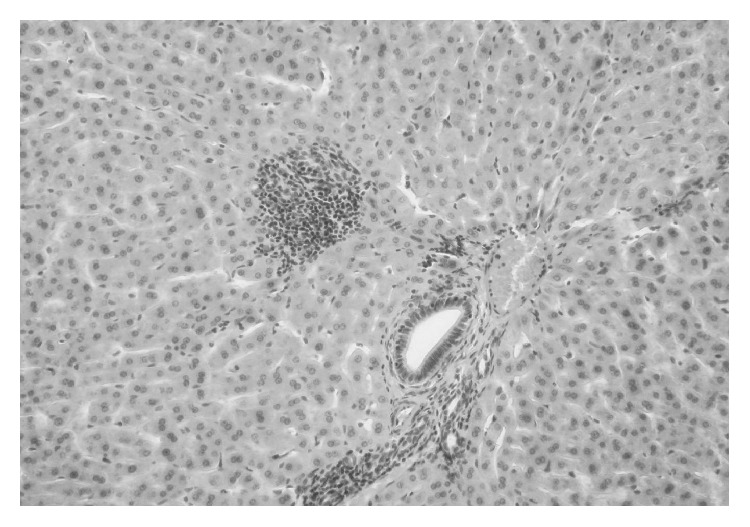
Liver tissue microphotography of oxidized rapeseed oil group. Fatty hepatocytes and focal necrotic lesions are present. H-E staining, 200x.

In animals from the RO group, retrogressive changes were observed in the form of focal necrotic lesions affecting hepatocytes in liver lobules in 2 rabbits and liver cell steatosis (Figure 6). All rabbits from that group presented mononuclear cells in their livers.

In the ROA group, only one rabbit showed necrotic lesions affecting hepatocytes in subcapsular area. Two rabbits had microinfiltrations in the hepatic triad area. All animals had an increased number of leukocytes in the lumen or in the vicinity of blood vessels, suggesting a decrease of immunological response.

In the ROG group, two of the six rabbits presented parenchymatous degeneration of hepatocytes in the central vein area; one had microinfiltrations in the hepatic triad area. No lesions were detected in hepatocytes or liver stoma of the remaining three rabbits.

**Figure 7:**
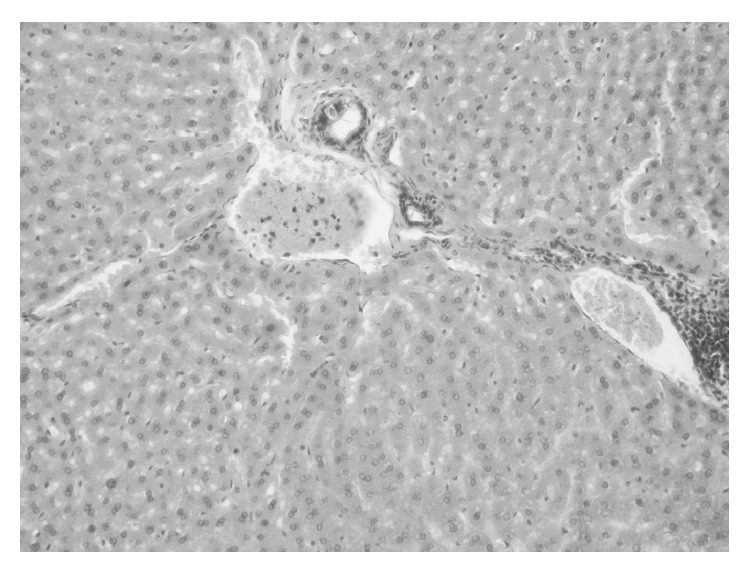
Liver tissue microphotography of oxidized olive oil group. Mononuclear cells infiltration around the triad area. H-E staining, 200x.

Retrogressive changes were detected in all OO rabbits, which presented focal or dispersed necrosis and extensive lobular steatosis. Only in one rabbit, steatosis affected the peripheral areas of the lobule. All of the lesions were accompanied by infiltrations from mononuclear cells (Figure 7).

Adding ALA to food enriched with oxidized olive oil (OOA group) resulted in the regression of steatosis and inflammatory lesions. Only one rabbit from each of those groups demonstrated infiltrations from mononuclear cells.

There were lesions of liver hepatocytes in three rabbits of the OOG group. In two cases there was swelling of hepatocytes and a third rabbit presented dispersed necrosis of the liver. The latter was accompanied by infiltrations from mononuclear cells in the hepatic triad area and local infiltrations between hepatocytes.

## 4. Discussion

Polyunsaturated fatty acids in vegetable oils are readily oxidized, decreasing their biological and nutritional properties. In the present study, the oxidation of rapeseed and olive oils caused a significant increase in the peroxide value, a decrease in the iodine value, increases of palmitic and oleic acids, and lower levels of linolic and linolenic acid (Table 1). Increased lipid peroxidation was confirmed by elevated MDA in liver tissue and TOS and LOO in blood serum of rabbits receiving oxidized oils. MDA concentration increased especially in the olive oil group while TOS and LOO concentrations increased especially in the rapeseed oil group.

As an indicator for assessing the intensity of the lipid peroxidation process, these biomarkers are correlated with the oxidative stability of oil and, in the case of oxidized oils, with the content of the primary and secondary oxidation products.

As early as 1975, Andia and Street reported an increase in the concentration of endogenous MDA resulting from a diet containing 15% of energy derived from fried oil [35]. Additionally, Izaki et al. noted increased levels of MDA in the liver of rats exposed to oxidized rapeseed oil derived from frying fish paste. They found that it was linked to increased content of arachidonic and docosahexaenoic acids in lipids as well as a marked drop of *α*-tocopherol in the oil [36]. Also, Tabatabaei et al. found that the consumption of vegetable oil oxidized for 48 hours at 180°C caused a significant increase in the MDA concentration in rat blood serum [37].

In one of our previous studies we reported that consumption of oxidized rapeseed oil induces lipid peroxidation and causes disturbed homeostasis in experimental animals. The study revealed that rabbits kept on high-fat diets enriched with RO oxidized at 120°C for one week had increased concentrations of MDA in blood serum and rabbit aorta homogenates. These changes were accompanied by increased concentration of 7-ketocholesterol, an endogenous indicator of free-radical oxidation of cholesterol [38, 39]. In other studies, we found that an HFD added with RO oxidized at 180°C for 6 hours caused disturbances in the activity of antioxidative enzymes due to intensified lipid peroxidation, confirmed by increased MDA levels both in blood serum and in liver [24, 25].

It appears that the increase of MDA concentration caused by an HFD enriched with thermally oxidized oils result from increased lipid peroxidation and probably from diet-induced autooxidation of native polyunsaturated fatty acids.

In the present study, the influence of *α*-lipoic acid and garlic lowered the concentration of indicators of oxidative stress. The MDA levels were lower in the treated, particularly in the OO, group. No such effect was noted in the RO group, but there was a slight decrease of the MDA concentration when the RO and ROG groups were compared (*P* = 0.075). This may indicate that ALA and garlic reduce lipid peroxidation by quenching peroxide radicals.

The ability of lipoic acid to neutralize free radicals is the basis for studies concerning its beneficial influence on pathologies that involve disruption of the prooxidative/antioxidative balance. In our previous studies conducted on rats on a high-fat diet with RO oxidized by heating to 180°C, addition of lipoic acid caused a decrease of the MDA concentration. It also resulted in normalization of the activities of the antioxidative enzymes peroxide dismutase (SOD) and glutathione peroxidase (GPx), as well as glucose-6-phosphate [24]. Similarly, the administration of garlic to rabbits fed a diet rich in thermally oxidized RO (120°C, 7 days) inhibited atherosclerotic changes in the aorta and seemed to be related to decreasing concentration of triacylglycerol in blood serum [18].

The results are in agreement with those of Metwally, who found a significant decrease of the MDA level in fish fed a garlic-containing diet [40]. In other studies, Augusti and Sheela showed that treatment of rats with S-allyl cysteine sulfoxide isolated from garlic reduced the extent of lipid peroxidation [41]. Schulz et al. reported that garlic supplementation resulted in decreased lipid peroxidation and enhanced antioxidant defense in the liver and kidneys of hamsters [42]. Thus, it may be concluded that garlic has an antioxidant effect by scavenging ROS and inhibiting lipid peroxidation.

Hagen et al. demonstrated that 2-week administration of 0.5% lipoic acid to rats resulted in reduction of the MDA concentration, with a simultaneous increase in the concentration of glutathione and ascorbic acid [43]. Shanmugarajan et al. found that administration of ALA increases the activity of SOD, catalase, and GPx and reductase in the blood of rats with an injured liver [44]. Cui et al. discovered that the addition of 0.1% lipoic acid to mice fed a diet rich in fats increased the expression of genes involved in the antioxidative protection [45]. Further studies of Cui et al. explained that lipoic acid also has a protective effect on the course of oxidative stress induced by a high-fat diet in the hippocampus area [46]. Those observations have been confirmed by Abdel-Hafeez et al., who demonstrated that the administration of *α*-lipoic acid caused increased concentration of glutathione, with a simultaneous reduction in the MDA concentration in animals with experimentally induced fibrosis of the liver [21].

There are other than MDA indicators of free radical production in the organism, such as total oxidant status (TOS) and lipid peroxides (LOO). TOS value informs about the total level of all peroxides contained in the examined material. It is a well-recognized marker, more sensitive and much more accurate than other individual products of lipid peroxidation measured separately. TOS is considered as one of the latest and quite stable markers of lipid peroxidation [47, 48]. In the present study, the TOS value increased significantly in both groups of animals receiving oxidized oils. The increase was more pronounced in the group receiving rapeseed oils. Lipid peroxides levels changed analogically. The highest concentration of LOO was reported also in the group receiving oxidized rapeseed oil. Less pronounced changes in the TOS values and LOO levels in the group receiving oxidized olive oil than in the group receiving rapeseed oil may be a result of higher oxidative stability of olive oil. Consequently, rapeseed oil is a source of a greater amount of exogenous peroxides than olive oil. These peroxides additively promote endogenous lipid peroxidation and may contribute to the higher TOS values and LOO levels observed in the rabbits receiving rapeseed oil (Table 1).

However, the additional dietary intake of ALA resulted in the decrease of oxidative stress biomarker concentrations, such as TOS value and the concentration of lipid peroxides (LOO) and MDA, in the groups receiving oxidized oils. Similar changes were reported in the groups receiving garlic. These results can be regarded as an evidence for the removal of peroxylipid radicals by ALA and garlic leading to the inhibition of lipid peroxidation propagation.

During this study, we found that the amount of 8OHdG was higher in the liver of rabbits subject to oxidative stress. The measured oxidative markers and histopathological examination confirmed the induced inflammatory condition of the liver, leading to mutagenic lesions in the course of long-term exposure. The presence of 8OHdG in the samples further confirms diet-induced oxidative stress.

These findings are in agreement with studies from other research teams. Irie et al. assessed the expression of the GGT gene and content of 8OHdG in liver sections, with simultaneous determination of the level of those indicators in blood serum in individuals with nonalcoholic steatohepatitis. The authors have proved that a high level of GGT activity in serum correlated positively with the level of 8OHdG in liver tissue, possibly confirming the participation of 8OHdG in the development of liver steatosis [49]. In the studies reported here, the groups of animals receiving oxidized oils had elevated activity of GGT in serum (data not shown), correlating to an elevated content of 8OHdG in liver tissue. Nomoto et al. confirmed the role of 8OHdG as a sensitive marker of the development of liver steatosis. In studies conducted on patients with diagnosed NASH, an increased amount of 8OHdG was found both in the cytoplasm and in hepatocyte nuclei [50]. Additionally, in a study concerning the role of oxidative stress in the development of regular and complicated NASH, Seki et al. reported increased concentrations of 4-HNE and 8OHdG [51].

In this study we observed that the addition of ALA acid to a diet rich in oxidized vegetable oils caused a significant reduction in 8OHdG in liver homogenates, particularly in the OOA group. This may confirm the beneficial role of ALA, resulting from its ability to attenuate inflammatory conditions. Suh et al. demonstrated that the administration of lipoic acid to rats under oxidative stress conditions significantly reduced the content of 8OHdG [52]. Kumar et al. also noted an increased amount of 8OHdG due to consumption of a high-cholesterol diet, which resulted in liver steatosis. At the same time, they emphasized the protective role of Liponate, lipoic and eicosapentaenoic acids in the prevention of experimental oxidative lesions of the liver, manifested by the normalization of 8OHdG levels [53]. It seems that the noted beneficial effect of ALA acid results from its ability to normalize the lipid profile, and mobilization of the enzymatic antioxidative defense system, as seen in the group of animals receiving HFD and ALA. To sum up, the significant decrease of the 8OHdG level in the groups receiving oxidized oils with the addition of both ALA and garlic can be considered as a strong evidence for their beneficial role against oxidative DNA damage.

A higher concentration of TNF*α* in blood serum as well as increased expression of mRNA for both* TNFα* and* IL-1α* genes may be an evidence of the chronic inflammation development in the group of animals received oxidized oils. Less pronounced changes in TNF*α* level and lower expression (at the threshold of statistical significance) of mRNA for* IL-1α* in animals receiving oxidized olive oil than in animals receiving oxidized rapeseed oil may be due to the higher oxidation stability of olive oil.

The addition of dietary ALA and garlic to both oxidized oils caused a decrease of the levels and expression of inflammatory cytokines (*TNFα*,* IL-1α*). This anti-inflammatory action of ALA and garlic appears to be an important factor protecting the organism against induction of oxidative lesions and cytotoxicity of oil oxidation products. This protective effect is confirmed also by a significant reduction of 8OHdG content in liver homogenates caused by the addition of dietary ALA and garlic.

## 5. Summary

The histopathological results and the concentrations of MDA and 8OHdG in liver homogenates, concentrations of TOS, LOO, and TNF*α* in blood serum, and* TNFα* and* IL-1α* gene expressions showed that HFD rich in oxidized rapeseed or olive oils underlies disorders of the liver leading to steatosis in rabbits.

The negative effects of HFD can be ameliorated by the simultaneous addition of dietary *α*-lipoic acid and garlic, the intake of which decreased liver lipid peroxidation intensity, free radical damage of DNA, and the development of nonalcoholic steatohepatitis. The beneficial role of ALA and garlic administration may be not only due to their antioxidative properties but also to the other mechanisms, such as anti-inflammatory and detoxification properties or inducing the synthesis of endogenous antioxidative enzymes.

## Figures and Tables

**Table 1:** Percentage content of fatty acids, peroxide value, and iodine value of nonoxidized and thermally oxidized oils.

Acids	Content of FA [%]			
	Rapeseed oil		Olive oil *extra virgin*	
	Nonoxidized	Oxidized	Nonoxidized	Oxidized
Palmitic (16 : 0)	4.8	7.1	11.5	12.3
Stearic (18 : 0)	0.95	1.2	2.0	2.2
Oleic (18 : 1) Ω-9	66.5	86	78.2	84.5
Linolic (18 : 2) Ω-6	17.4	2.5	8.7	5.8
Linolenic (18 : 3) Ω-3	8.5	1.2	2.3	0.5
Peroxide value	0.47	11.20	9.80	12.0
Iodine value	104.4	102.92	94.7	9.90

**Table 2:** Statistical comparison of the MDA and 8OHdG levels in rabbits' liver in all study groups, with or without addition of *α*-lipoic acid and/or garlic.

Group	Additive	*N*	MDA [*μ*mol/g protein]		8OHdG	
			Mean	SD	Mean	SD
C	—	10	0.309	0.05	0	0
C	ALA	8	0.348	0.142	0.016	0.012
C	Garlic	9	0.313	0.097	0.004	0.001
RO	—	8	0.467	0.119	0.052	0.014
RO	ALA	9	0.374	0.11	0.052	0.014
RO	Garlic	8	0.351	0.06	0.028	0.007
OO	—	8	0.532	0.091	0.115	0.061
OO	ALA	6	0.373	0.022	0.071	0.028
OO	Garlic	7	0.356	0.037	0.008	0.002

**Table 3:** Post hoc tests of MDA and 8OHdG levels in rabbits' liver in all groups.

Group	Additive	MDA		8OHdG	
		ALA	Garlic	ALA	Garlic
C	—	0.717	0.995	0.177	0.731
	ALA	—	0.768	—	0.311

OO	—	<0.001	<0.001	<0.001	<0.001
	ALA	—	0.881	—	<0.001

RO	—	0.176	0.075	1.000	<0.05
	ALA	—	0.890	—	<0.05

**Table 4:** Statistical comparison of the TOS, LOO, and TNF*α* in blood serum, TNF*α*, and IL-1*α* gene expressions [RQ] in all study groups, with or without addition of *α*-lipoic acid and/or garlic.

Group	Additive	*N*	TOS [*μ*mol/L]		LOO [*μ*mol/L]		TNF*α* [pg/mL]		TNF*α* [RQ]		IL-1*α* [RQ]	
			Mean	SD	Mean	SD	Mean	SD	Mean	SD	Mean	SD
C	—	10	14.71	1.31	3.96	0.73	25.03	6.11	0.00	0.00	0.00	0.00
C	ALA	8	12.33	4.22	4.83	1.79	22.47	8.77	2.21	0.57	0.53	0.35
C	Garlic	9	13.45	2.44	5.11	1.71	38.02	12.39	1.47	1.21	0.55	0.37
RO	—	8	39.19	24.21	18.70	5.38	71.10	18.96	13.36	2.50	14.69	1.24
RO	ALA	9	15.47	6.51	9.86	4.10	52.45	15.14	2.45	1.43	2.46	2.70
RO	Garlic	8	15.17	4.34	9.15	2.25	50.88	15.77	0.82	0.93	2.18	4.23
OO	—	8	21.37	4.21	11.99	1.94	47.50	11.36	12.74	3.51	12.14	1.45
OO	ALA	6	13.36	2.05	8.54	0.99	37.39	21.98	5.56	3.17	2.78	0.54
OO	Garlic	7	14.30	2.40	9.78	0.77	27.37	9.79	1.80	0.89	4.85	0.72

**Table 5:** Post hoc tests of TOS, LOO, and TNF*α* in blood serum, *TNFα*, and *IL-1α* gene expressions [RQ] in all groups.

Group	Additive	TOS		LOO		TNF*α*		TNF*α* [RQ]		IL-1*α* [RQ]	
		ALA	Garlic	ALA	Garlic	ALA	Garlic	ALA	Garlic	ALA	Garlic
C	—	0,124	0,656	0,435	0,243	0,831	<0,05	<0,001	<0,01	<0,01	<0,01
	ALA	—	0,490	—	0,915	—	<0,01	—	0,197	—	0,994

OO	—	<0,001	<0,001	<0,01	<0,05	0,474	<0,05	<0,01	<0,001	<0,001	<0,001
	ALA	—	0,793	—	0,300	—	0,479	—	0,123	—	<0,05

RO	—	<0,01	<0,01	<0,01	<0,001	0,065	<0,05	<0,001	<0,001	<0,001	<0,001
	ALA	—	0,999	—	0,928	—	0,979	—	0,270	—	0,985

## Abbreviations

Ct: Threshold cycle
2′dG: 2′-Deoxyguanosine
GAPDH: Glyceraldehyde-3-phosphate dehydrogenase gene
4HNE: 4-Hydroxynonenal
8OHdG: 8-Hydroxy-2′-deoxyguanosine
ALA: *α*-Lipoic acid
BD: Basal diet
C: Control group
FA: Fatty acids
G: Garlic
GGT: Gamma-glutamyltransferase
GPx: Glutathione peroxidase
H-E: Hematoxylin-eosin
HFD: High-fat diet
HPLC: High pressure liquid chromatography
IL-1*α*: Interleukin 1 alpha
IV: Iodine value
LOO: Lipid peroxides
MDA: Malondialdehyde
NASH: Nonalcoholic steatohepatitis
OOA: Oxidized olive oil with *α*-lipoic acid
OOG: Oxidized olive oil with garlic
PUFA: Polyunsaturated fatty acids
PV: Peroxide value
(qRT)-PCR: Quantitative Reverse Transcription Polymerase Chain Reaction
RO: Oxidized rapeseed oil
ROA: Oxidized rapeseed oil with *α*-lipoic acid
ROG: Oxidized rapeseed oil with garlic
ROS: Reactive oxygen species
RQ: Relative gene expression levels
SOD: Superoxide dismutase
TBARS: Thiobarbituric acid-reactive substances
TNF*α*: Tumor necrosis factor alpha.

## References

[1] Balcerczyk A., Bartosz G. (2003). Thiols are main determinants of total antioxidant capacity of cellular homogenates. *Free Radical Research*.

[2] Bandyopadhyay U., Das D., Banerjee R. K. (1999). Reactive oxygen species: oxidative damage and pathogenesis. *Current Science*.

[3] Dotan Y., Lichtenberg D., Pinchuk I. (2004). Lipid peroxidation cannot be used as a universal criterion of oxidative stress. *Progress in Lipid Research*.

[4] Marnett L. J. (1999). Lipid peroxidation-DNA damage by malondialdehyde. *Mutation Research: Fundamental and Molecular Mechanisms of Mutagenesis*.

[5] Gay C. A., Gebicki J. M. (2003). Measurement of protein and lipid hydroperoxides in biological systems by the ferric-xylenol orange method. *Analytical Biochemistry*.

[6] Halliwell B., Gutteridge J. M. C. (2000). *Free Radicals in Biology and Medicine*.

[7] Lindschinger M., Nadlinger K., Adelwöhrer N. (2004). Oxidative stress: potential of distinct peroxide determination systems. *Clinical Chemistry and Laboratory Medicine*.

[8] O'Brien P. J. (2000). Peroxidases. *Chemico-Biological Interactions*.

[9] Kasai H. (1997). Analysis of a form of oxidative DNA damage, 8-hydroxy-2′-deoxyguanosine, as a marker of cellular oxidative stress during carcinogenesis. *Mutation Research—Reviews in Mutation Research*.

[10] Cheng K. C., Cahill D. S., Kasai H., Nishimura S., Loeb L. A. (1992). 8-hydroxyguanine, an abundant form of oxidative damage, causes G→T and A→C substitutions. *Journal of Molecular Biology*.

[11] Kasai H. (2002). Chemistry-based studies on oxidative DNA damage: formation, repair, and mutagenesis. *Free Radical Biology and Medicine*.

[12] Gannett P. M., Sura T. P. (1993). Base pairing of 8-oxoguanosine and 8-oxo-2′-deoxyguanosine with 2′-deoxyadenosine, 2′-deoxycytosine, 2′-deoxyguanosine, and thymidine. *Chemical Research in Toxicology*.

[13] Valko M., Leibfritz D., Moncol J., Cronin M. T. D., Mazur M., Telser J. (2007). Free radicals and antioxidants in normal physiological functions and human disease. *International Journal of Biochemistry and Cell Biology*.

[14] Bolner A., Pilleri M., De Riva V., Nordera G. P. (2011). Plasma and urinary HPLC-ED determination of the ratio of 8-OHdG/2-dG in Parkinson's disease. *Clinical Laboratory*.

[15] Gmitterova K., Heinemann U., Gawinecka J. (2009). 8-OHdG in cerebrospinal fluid as a marker of oxidative stress in various neurodegenerative diseases. *Neurodegenerative Diseases*.

[16] Lunec J., Herbert K., Blount S., Griffiths H. R., Emery P. (1994). 8-hydroxydeoxyguanosine: a marker of oxidative DNA damage in systemic lupus erythematosus. *FEBS Letters*.

[17] Wu L. L., Chiou C.-C., Chang P.-Y., Wu J. T. (2004). Urinary 8-OHdG: a marker of oxidative stress to DNA and a risk factor for cancer, atherosclerosis and diabetics. *Clinica Chimica Acta*.

[18] Zalejska-Fiolka J., Kasperczyk A., Kasperczyk S., Błaszczyk U., Birkner E. (2007). Effect of garlic supplementation on erythrocytes antioxidant parameters, lipid peroxidation, and atherosclerotic plaque formation process in oxidized oil-fed rabbits. *Biological Trace Element Research*.

[19] Huk-Kolega H., Skibska B., Kleniewska P., Piechota A., Michalski Ł., Gorąca A. (2011). Rola kwasu liponowego w zdrowiu i chorobie. *Polski Merkuriusz Lekarski*.

[20] Malińska D., Winiarska K. (2005). Lipoic acid: characteristics and therapeutic application. *Postępy Higieny i Medycyny Doświadczalnej*.

[21] Abdel-Hafeez E. H., Ahmad A. K., Abdulla A. M., Aabdel-Wahab S., Mosalem F. A. (2012). Therapeutic effect of alpha lipoic acid combined with praziquantel on liver fibrosis induced by Schistosoma mansoni challenged mice. *Parasitology Research*.

[22] Kaya M., Yildirim C. H., Kosemehmetoglu K. (2012). Alpha-lipoic acid reduces peridural fibrosis after laminectomy of lumbar vertebrae in rabbits. *Acta Neurochirurgica*.

[23] Tian Y.-F., Hsieh C.-H., Hsieh Y.-J., Chen Y.-T., Peng Y.-J., Hsieh P.-S. (2012). *α*-lipoic acid prevents mild portal endotoxaemia-induced hepatic inflammation and *β* cell dysfunction. *European Journal of Clinical Investigation*.

[24] Zalejska-Fiolka J., Wielkoszyński T., Kasperczyk S., Kasperczyk A., Birkner E. (2010). Effects of oxidized cooking oil and *α*-lipoic acid on liver antioxidants: enzyme activities and lipid peroxidation in rats fed a high fat diet. *Biological Trace Element Research*.

[25] Zalejska-Fiolka J., Wielkoszyński T., Kasperczyk S., Kasperczyk A., Birkner E. (2012). Effects of oxidized cooking oil and *α*-lipoic acid on blood antioxidants: enzyme activities and lipid peroxidation in rats fed a high-fat diet. *Biological Trace Element Research*.

[26] Banerjee S. K., Mukherjee P. K., Maulik S. K. (2003). Garlic as an antioxidant: the good, the bad and the ugly. *Phytotherapy Research*.

[28] Erel O. (2005). A new automated colorimetric method for measuring total oxidant status. *Clinical Biochemistry*.

[29] Södergren E., Nourooz-Zadeh J., Berglund L., Vessby B. (1998). Re-evaluation of the ferrous oxidation in xylenol orange assay for the measurement of plasma lipid hydroperoxides. *Journal of Biochemical and Biophysical Methods*.

[30] Ohkawa H., Ohishi N., Yagi K. (1979). Assay for lipid peroxides in animal tissues by thiobarbituric acid reaction. *Analytical Biochemistry*.

[31] Lowry O. H., Rosenbrough N. J., Farr A. L., Randall R. J. (1951). Protein measurement with the Folin phenol reagent. *The Journal of Biological Chemistry*.

[32] Foksinski M., Bialkowski K., Skiba M., Ponikowska I., Szmurlo W., Olinski R. (1999). Evaluation of 8-oxodeoxyguanosine, typical oxidative DNA damage, in lymphocytes of ozone-treated arteriosclerotic patients. *Mutation Research*.

[33] Shigenaga M. K., Gimeno C. J., Ames B. N. (1989). Urinary 8-hydroxy-2′-deoxyguanosine as a biological marker of in vivo oxidative DNA damage. *Proceedings of the National Academy of Sciences of the United States of America*.

[34] Zawistowski S. (1986). *Technika histologiczna, histologia oraz podstawy histopatologii*.

[35] Andia A. M. G., Street J. C. (1975). Dietary induction of hepatic microsomal enzymes by thermally oxidized fats. *Journal of Agricultural and Food Chemistry*.

[36] Izaki Y., Yoshikawa S., Uchiyama M. (1984). Effect of ingestion of thermally oxidized frying oil on peroxidative criteria in rats. *Lipids*.

[37] Tabatabaei N., Jamalian J., Owji A. A., Ramezani R., Karbalaie N., Rajaeifard A. R. (2008). Effects of dietary selenium supplementation on serum and liver selenium, serum malondialdehyde and liver glutathione peroxidase activity in rats consuming thermally oxidized sunflower oil. *Food and Chemical Toxicology*.

[38] Zalejska-Fiolka J., Kasperczyk A., Kasperczyk S., Błaszczyk U., Birkner E. (2007). Effect of oxidised rapeseed oil with garlic on the concentration of 7-ketocholesterol, malondialdehyde, and free fatty acids in hypercholesterolaemic rabbits. *Bulletin of the Veterinary Institute in Pulawy*.

[39] Zalejska-Fiolka J., Kasperczyk S., Kasperczyk A. (2004). Influence of oxidated vegetable oil and garlic extract upon the development of experimental atherosclerosis in rabbits. *Bulletin of the Veterinary Institute in Pulawy*.

[40] Metwally M. A. A. (2009). Effects of garlic (*Allium sativum*) on some antioxidant activities in Tilapia Nilotica (*Oreochromis niloticus*). *World Journal of Fish and Marine Sciences*.

[41] Augusti K. T., Sheela C. G. (1996). Antiperoxide effect of S-allyl cysteine sulfoxide, an insulin secretagogue, in diabetic rats. *Experientia*.

[42] Schulz V. R., Hansel V. T., Blumenthal M. (2004). *Rational Phytotherapy: A Physician's Guide*.

[43] Hagen T. M., Ingersoll R. T., Lykkesfeldt J. (1999). (R)-*α*-lipoic acid-supplemented old rats have improved mitochondrial function, decreased oxidative damage, and increased metabolic rate. *The FASEB Journal*.

[44] Shanmugarajan T. S., Sivaraman D., Somasundaram I. (2008). Influence of alpha lipoic acid on antioxidant status in D-galactosamine-induced hepatic injury. *Toxicology and Industrial Health*.

[45] Cui J., Xiao Y., Shi Y.-H., Wang B., Le G.-W. (2012). Lipoic acid attenuates high-fat-diet-induced oxidative stress and B-cell-related immune depression. *Nutrition*.

[46] Cui Y., Shu Y., Zhu Y., Shi Y., Le G. (2012). High-fat diets impair spatial learning of mice in the Y-maze paradigm: ameliorative potential of *α*-lipoic acid. *Journal of Medicinal Food*.

[47] Bartosz G. (2008). *Druga Twarz Tlenu—The Second Face of Oxygen*.

[48] Rice Evans C. A., Diplock A. T. (1991). *Symons MCR: Techniques in Free Radical Reseach*.

[49] Irie M., Sohda T., Iwata K. (2012). Levels of the oxidative stress marker *γ*-glutamyltranspeptidase at different stages of nonalcoholic fatty liver disease. *Journal of International Medical Research*.

[50] Nomoto K., Tsuneyama K., Takahashi H., Murai Y., Takano Y. (2008). Cytoplasmic fine granular expression of 8-hydroxydeoxyguanosine reflects early mitochondrial oxidative DNA damage in nonalcoholic fatty liver disease. *Applied Immunohistochemistry and Molecular Morphology*.

[51] Seki S., Kitada T., Sakaguchi H. (2005). Clinicopathological significance of oxidative cellular damage in non-alcoholic fatty liver diseases. *Hepatology Research*.

[52] Suh J. H., Shigeno E. T., Morrow J. D. (2001). Oxidative stress in the aging rat heart is reversed by dietary supplementation with (R)-*α*-lipoic acid. *The FASEB Journal*.

[53] Kumar S. A., Sudhahar V., Varalakshmi P. (2006). Protective role of eicosapentaenoate-lipoate (EPA-LA) derivative in combating oxidative hepatocellular injury in hypercholesterolemic atherogenesis. *Atherosclerosis*.

